# BODIPY–BODIPY dyad: assessing the potential as a viscometer for molecular and ionic liquids†
†Electronic supplementary information (ESI) available: Details on the synthesis and purification of BODIPY dyes and ILs, sample preparations and physical properties of ILs as well as spectral data. See DOI: 10.1039/c4ra09757b



**DOI:** 10.1039/c4ra09757b

**Published:** 2015-02-17

**Authors:** Joseph D. Kimball, Sangram Raut, Laramie P. Jameson, Nicholas W. Smith, Zygmunt Gryczynski, Sergei V. Dzyuba

**Affiliations:** a Department of Physics and Astronomy , Texas Christian University , Fort Worth , TX 76129 , USA . Email: z.gryczynski@tcu.edu ; Fax: +1 817 257 7742 ; Tel: +1 817 257 4209; b Department of Chemistry , Texas Christian University , Fort Worth , TX 76129 , USA . Email: s.dzyuba@tcu.edu ; Fax: +1 817 257 5851 ; Tel: +1 817 257 6218

## Abstract

A symmetrical BODIPY–BODIPY dyad with a diyne linker was prepared in two steps; the lifetime decay of this rotor appeared to correlate with the viscosity of the media, thus making this dyad a suitable small molecule viscometer for molecular solvents. The potential of using the rotor to probe the viscosity of ionic liquids was also investigated.

## Abstract 2

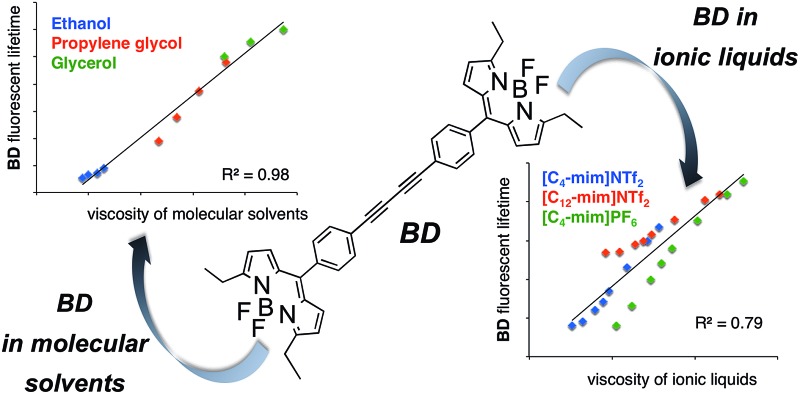
The fluorescent lifetimes of the BODIPY–BODIPY dyad appear to correlate with the viscosity of the media, thus making this rotor a suitable small molecule viscometer.

BODIPY dyes are versatile fluorophores that have received a significant amount of attention due to their wide-spread applications in chemical, biological, and materials sciences. Importantly, the photophysical properties of these dyes can be tuned *via* structural modifications of the boron-dipyrromethene scaffold.^1–3^ As such, BODIPY dyes offer a number of advantages over other types of fluorescent dyes, owing to the relatively facile synthetic manipulations that allow for the incorporation of various moieties in a fairly straightforward and modular manner, although it is not always very efficient and/or economical.

Viscosity is a fundamental property of fluid media, and therefore evaluating the viscosity in various environments using molecular rotors is an attractive area of modern research with numerous applications in chemistry, biology and drug discovery. Arguably, due to the sensitivity of fluorescence, fluorescent lifetimes as well as the potential for high-throughput screening and imaging, the use of fluorescent probes as molecular viscometers, might provide ample opportunities and advances.^4^

Several BODIPY-based viscometers have been recently reported. Typically, the rotor-moiety is attached at the *meso*-position of the BODIPY scaffold, and no substituents are present in the 1- and 7-positions to allow for a rotation around the single C–C bond (Fig. 1, **A**).^5^ In general, an increase in viscosity should suppress the rotation of the substituents around the BODIPY core in the exited state, and as a result suppress the non-radiative decay, which will lead to an increase in emission intensity, quantum yield, and lifetime.^5–11^ It could be assumed that the photophysical properties of BODIPY dyes, lifetimes in particular, are insensitive to variations in the polarity and pH of the media, thus making these dyes viable for monitoring viscosity changes within different types of media. Specifically, the lifetimes of BODIPY viscometers (typically in the ns range) showed a linear correlation with the viscosity in the 15–1000 mPa s range. The main application of these rotors has been the evaluation of viscosity of molecular solvents as well as cellular environments.^5–11^ Several examples for using BODIPY-based rotors as fluorescent pressure sensors have also been reported.^12,13^

**Fig. 1:**
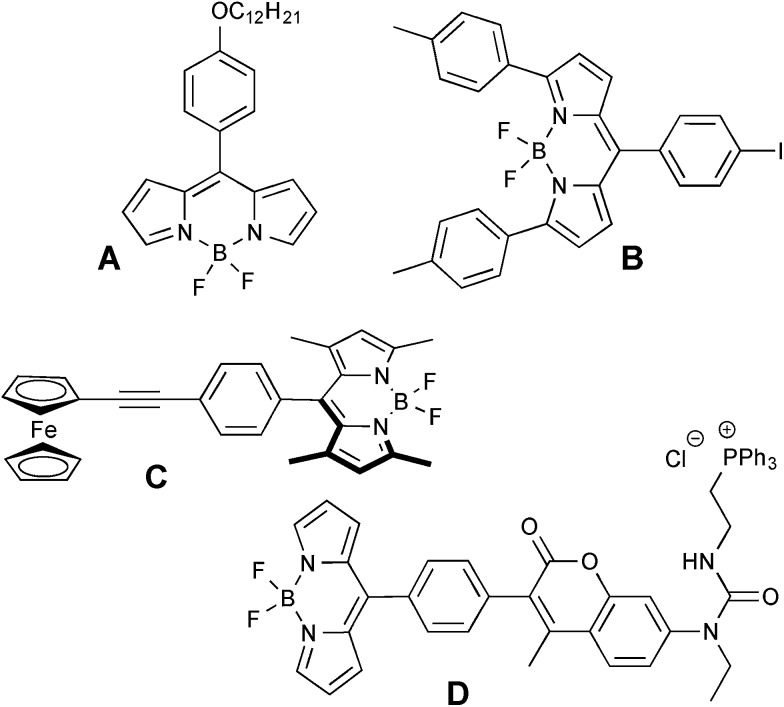
Some representative examples of BODIPY based viscometers; **A** – ref. 7; **B** – ref. 15; **C** – ref. 16; **D** – ref. 17.

Additionally, temperature-dependent changes in the microviscosity of poly(*N*-isopropylacrylamide-4-chloromethylstyrene) polymers were recorded by covalently attaching a BODIPY fluorophore to the polymer's side chain.^14^ Also, an effect of the so-called remote substituents, *i.e.*, aromatic (phenyl and tolyl) moieties in the 3- and 5-positions of the BODIPY motif (Fig. 1, **B**) on the ability to respond to changes in the media's viscosity was evaluated in a series of homologous, straight chain alcohols with increasing viscosity (*ca.* 0.5–10 mPa s).^15^

Only a few examples of BODIPY dyads as molecular viscometers have been reported. Specifically, the fluorescence intensity as well as quantum yield of a chimeric viscometer, featuring a BODIPY–ferrocene motif (Fig. 1, **C**), exhibited a linear correlation with the viscosity of THF–ethylene glycol mixtures.^16^ Concurrently, the fluorescence lifetimes increased from 2.3 ns to 3.7 ns as the concentration of ethylene glycol increased from 10 to 90%. Furthermore, a triphenylphosphonium-containing coumarin-BODIPY viscometer (Fig. 1, **D**) was shown to report changes in viscosity within mitochondria.^17^ Recently, a chimeric BODIPY-Nile Red probe that is capable of reporting on both polarity and viscosity of the specific media was also developed.^18^

Here, we present a structurally simple and easily accessible symmetric BODIPY–BODIPY dye suitable for probing a wide range of viscosities of both molecular and ionic solvents. Both BODIPY (**1**) and alkyne-BODIPY (**2**) were prepared according to either a conventional synthesis^19^ or by a recently developed mechanochemical procedure,^20^ and the subsequent dimerization of dye **2** was accomplished under Glaiser-type conditions^21,22^ to furnish **BD** in a moderate yield (Scheme 1).†

**Scheme 1:**
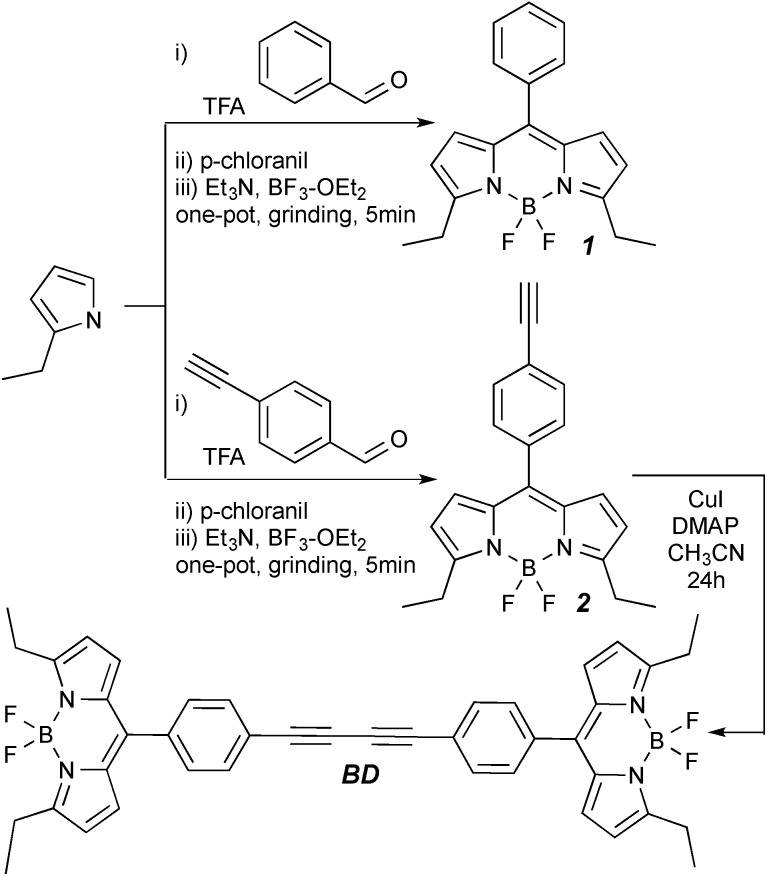
Synthesis of BODIPY based viscometers used in this work.

The fluorescence spectra of dyes **1**, **2**, and **BD** in ethanol, propylene glycol, and glycerol revealed that the position of neither the absorption nor the emission maxima (Fig. S1–S3 and Table S1†) were greatly affected by the viscosity of the media (Table S2†). On the other hand, the lifetime of dyes **2** and **BD** appeared to change notably (Fig. S4 and Table S1†). Specifically, the fluorescence intensity decay times for dye **2** increased from *τ*_int_ = 0.550 ns in ethanol (*η* = 1.2 mPa s) to *τ*_int_ = 3.060 ns in glycerol (*η* = 1457 mPa s). In the case of **BD**, the change was even more pronounced with *τ*_int_ = 0.326 ns in ethanol as compared to *τ*_int_ = 4.500 ns in glycerol. Although dyes **1** showed only a marginal (*ca*. 3-fold) increase in the fluorescent lifetime within the given viscosity range (Table S1†), this change is worth noting, primarily in view of a very simple structure of **1**, where the rotor was realized by a simple rotation of the phenyl group in the *meso*-position. Owing to the large change in fluorescence lifetime over the given viscosity range (1.2 to 1457 mPa s), *i.e.*, 13.8-fold for **BD** as compared to 5.6-fold for **2**, and 3.3-fold for **1**, we chose to further explore the possibility of using **BD** as a potential molecular viscometer.

Furthermore, the effect of media's temperature on the fluorescence lifetime of **BD** was studied in ethanol, propylene glycol, and glycerol (Tables S2 and S3†) and showed an apparent linear correlation between the viscosity of the solvents and the fluorescence lifetime of **BD** (Fig. 2). This observed correlation between the fluorescent lifetimes and the viscosity of the media (which is changed by both altering the nature of the media and/or altering the temperature of the media) should be indicative of the fact that viscosity is indeed the main factor that modulates the conformation preference of **BD**. It should also be pointed out that the fluorescence intensity decays exhibited a multi-exponential behaviour as a function of the identity of the solvent as well as temperature (Table S4†).

**Fig. 2:**
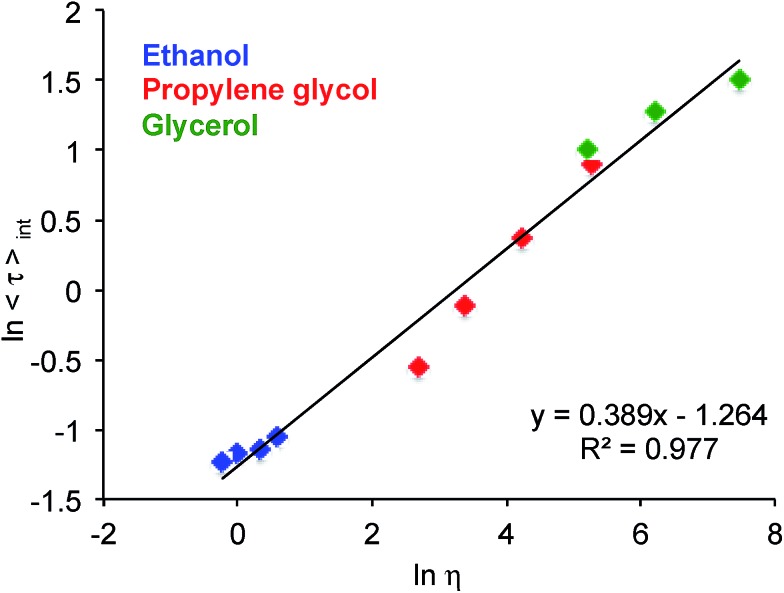
Effect of media's viscosity on the lifetime of **BD** in ethanol, propylene glycol and glycerol in a 5–50 °C (Δ*T* = 15 °C) temperature range.

Collectively, these results suggested that **BD** could potentially be used as a simple, minimalistic dyad to monitor viscosity of the molecular media.

Next, in order to explore other types of environments, we decided to probe the potential of **BD** as a molecular viscometer in ionic media, *i.e.*, room-temperature ionic liquids (ILs), and 1,3-dialkylmidazolium ILs are among the most widely used and studied ILs.^23^ These solvents have found numerous applications, and they could be viewed as customizable materials since their physical properties, including viscosity, can be tuned *via* structural modifications of the cationic and anionic counterparts.^23–26^ The viscosity of the 1-alkyl-3-methylimidazolium ILs can be modulated over a wide range by simply adjusting the length of the alkyl chains, as well as the identity of the anion.^27–31^

Importantly, on the structural level, neat ILs could be viewed as heterogeneous, supramolecular polymer-like assemblies (or nanostructured domains) as opposed to molecular solvents that are homogeneous fluids.^32–37^ This heterogeneity was also demonstrated to be crucial in controlling the outcome of several organic reactions performed in ILs.^38,39^

Here, we investigated the behaviour of **BD** in several ILs, namely [C_4_-mim]PF_4_, [C_4_-mim]NTf_2_, and [C_12_-mim]NTf_2_ (Fig. 3). These ILs were chosen based on (i) their broad viscosity range (78 to 435 mPa s at 20 °C, see Table S4†), which complements that of the molecular solvents (Table S2†), and (ii) their largely non-hygroscopic nature (as compared to more hydrophilic ILs, such as NO_3_- and BF_4_-containing ILs). This was expected to facilitate the handling and manipulation of the ILs during the measurements.

**Fig. 3:**
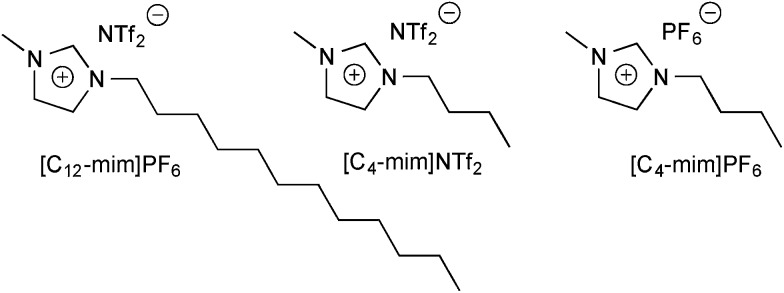
Structures of ILs used in this work.

Similar to molecular solvents (Fig. S3†), the absorption and emission spectra of **BD** were virtually unchanged in all three ILs (Fig. S5 and Table S5†), while the fluorescent lifetimes of **BD** increased as the viscosity of the ILs increased (Fig. S6 and Table S6†). In addition, multi-exponential fluorescence decays were noted for **BD** as well (Table S6†).

However, when the fluorescence lifetimes of **BD** in ILs were plotted as a function of ILs viscosity a dramatically different relationship from that observed in molecular solvents (Fig. 2) was observed (Fig. 4). Specifically, for the two ILs with the same cation, *i.e.*, [C_4_-mim]PF_6_ and [C_4_-mim]NTf_2_ the slopes were 0.498 and 0.696, respectively, whereas for the ILs with the same anion, *i.e.*, [C_4_-mim]NTf_2_ and [C_12_-mim]NTf_2_ the slopes were 0.696 and 0.249, respectively (Fig. 4). It could be argued that a different slope in the viscosity *versus* lifetime dependence is attributed to a unique solute–solvent interaction.

**Fig. 4:**
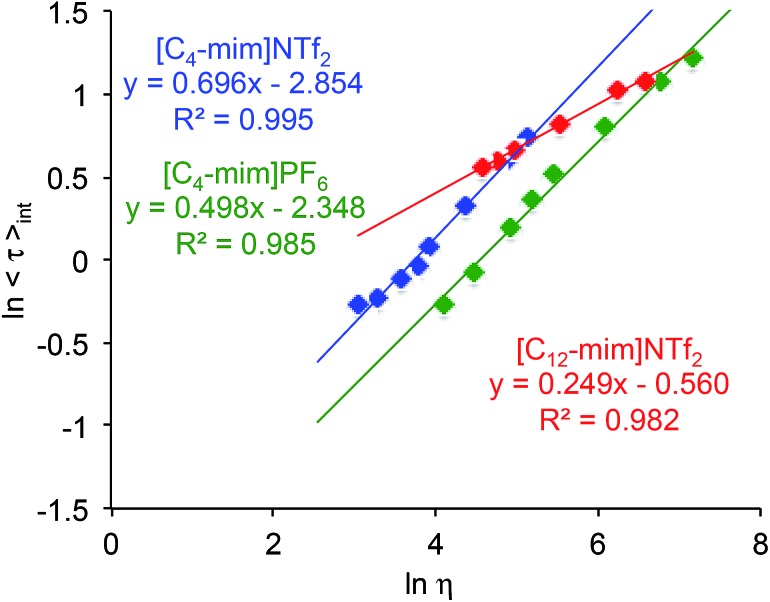
Effect of media's viscosity on the lifetime of **BD** in [C_4_-mim]NTf_2_, [C_4_-mim]PF_6_ and [C_12_-mim]NTf_2_ in a 5–60 °C (Δ*T* = 10 °C) temperature range.

Alternatively, it could also be proposed that unique behaviour of **BD** in each IL might be due to distinct nanostructured assemblies of these ILs. Considering that in molecular solvents, *i.e.*, ethanol, propylene glycol, and glycerol, within similar temperature range (5–60 °C), a linear correlation for all solvents was observed (Fig. 2), which could indicate that no specific interactions between the solvent and **BD** exist and/or no heterogeneous domains exist in these solvents. It is also worth noting that the spectroscopic behaviour of an ionic porphyrin-based viscometer in molecular solvents was quite different from that in ILs.^40^ Although these results suggested that generalizations regarding the viscosity of ILs using molecular viscometers should be carried out with caution, due to the unique nature of this type of media, it might be possible, in principle, to use such molecules to probe the nature of heterogeneous IL domains and/or assess how different the IL domains could be (in terms of the heterogeneity).

## Conclusions

**BD** was prepared in two steps from commercially available starting materials, and it appeared to be a viable molecular viscometer for molecular solvents. The assessment of the viscosity of ILs using **BD** was less straightforward, most likely due to specific-solvent interactions and/or the unique aggregation state (or heterogeneity) of specific ILs. Nonetheless, **BD** appears to be a viable viscometer for monitoring viscosity changes of molecular solvents; based on our recent study, **BD** might also be suitable for probing viscosity of cellular and membrane-like environments.^41^
