# Supplementary material for: BODIPY–BODIPY dyad: assessing the potential as a viscometer for molecular and ionic liquids
Source: RSC Adv. 2015 Feb 17;5(25):19508–11. doi: 10.1039/c4ra09757b (PMC4381807; doi:10.1039/c4ra09757b)
Supplement: Supplementary file 1 [file RA-005-C4RA09757B-s001.pdf]

Electronic Supplementary Material (ESI) for RSC Advances.
This journal is © The Royal Society of Chemistry 2015

SUPPORTING INFORMATION

BODIPY-BODIPY dyad: assessing the potential as a viscometer for molecular and ionic liquids

Joseph D. Kimball,[a] Sangram Raut,[a] Laramie P. Jameson,[b] Nicholas W. Smith,[b] Zygmunt Gryczynski*[a] and Sergei V. Dzyuba*[b]

[a] *Department of Physics and Astronomy, Texas Christian University, Fort Worth, TX 76129, USA. Fax: +1 817 257 7742; Tel: +1 817 257 4209; E-mail: z.gryczynski@tcu.edu*

[b] *Department of Chemistry, Texas Christian University, Fort Worth, TX 76129, USA. Fax: +1 817 257 5851; Tel: +1 817 257 6218; E-mail: s.dzyuba@tcu.edu*

TABLE OF CONTENTS

Materials and methods…………………………………………..……..…………………… 2

Synthesis and characterization of BODIPY dyes…..…………………………………… 2

Synthesis and characterization of ionic liquids…...………………..…………………....... 3

Spectroscopic measurements…………………………………………………………… 4

Table S1 Photophysical properties (absorption maxima, emission maxima, fluorescence lifetime, and quantum yield) of dyes **1**, **2** and **BD** in molecular solvents at 20°C ……….. 5

Figure S2 Effect of temperature on the viscosity of molecular solvents……………..... 5

Table S3 Effect of temperature on fluorescence lifetime of **BD** in molecular solvents. 6

Table S4 Effect of temperature on viscosity of ionic liquids…………………………… 7

Table S5 Photophysical properties (absorption maxima, emission maxima, fluorescence lifetime, and quantum yield) of **BD** in ionic liquids at 20°C…… 7

Table S6 Effect of temperature on fluorescence lifetime of **BD** in ionic liquids……... 8

Figure 1S Absorption (left) and emission (right) spectra of dye **1** in molecular solvents……………..………………………………………………………… 10

Figure S2 Absorption (left) and emission (right) spectra of dye **2** in molecular solvents……………..………………………………………………………… 10

Figure S3 Absorption (left) and emission (right) spectra of dye **BD** in molecular solvents…………..…………………………………………………………… 11

Figure S4 Fluorescence intensity decays for **BD** dye in molecular solvents at 20°C... 11

Figure S5 Absorption (left) and emission (right) spectra of dye **BD** in ionic liquids….. 12

Figure S6 Fluorescence intensity decays for **BD** dye in ionic liquids at 20°C.………. 12

Figure S7 Temperature-dependent fluorescence intensity decays of **BD** in ionic liquids……..………………………………………………………………….... 13

References……………………………………………………………………….…..... 14

Materials and Methods

All chemicals and solvents were from commercial sources, they were of highest grade possible, and were used as received. ^{1}H NMR, ^{13}C NMR, and ^{19}F spectra were recorded on a Varian (300 MHz) or Bruker (400 MHz) spectrometers; the chemical shifts are reported in ppm (δ) downfield from tetramethylsilane in $CDCl_3$. High-resolution mass spectra (HRMS) were measured under EI, direct probe conditions at the Mass Spectrometry facility at the University of Notre Dame. Viscosity of ILs and molecular solvents as well as their mixtures was measured using Anton-Paar Lovis 4500M microviscometer. Following the viscosity measurements, water content of ILs and molecular solvents was measured using Aquamax KF coulometric titrator from GRS Scientific according to manufacturer provided protocols, using 0.4 or 0.5 ml of sample.

Synthesis and characterization of BODIPY dyes

Dyes **1** and **2** were prepared according to literature procedures.[1]

BODIPY-BODIPY dyad (**BD**) was prepared according to a modified typical procedure for Glaiser coupling. Briefly, a screw cap vial equipped with a stirring bar was charged with dye **2** (30.0 mg, 0.086 mmol), dimethylaminopyridine, DMAP (22.0 mg, 0.180 mmol), CuI (18.0 mg, 0.095 mmol), followed by the addition of CH_3CN (1.0 ml). The reaction mixture was stirred for 48 h at room temperature, before being diluted with CH_2Cl_2 (15 ml), and washed sequentially with 1 M HCl solution (2 x 10 ml) and brine (10 ml). Volatiles were removed in vacuo, and the resulting residue subjected to column chromatography (silica gel; CH_3Cl eluent) to give **BD** as a red solid (10.8 mg, 35 % yield).

^{1}H NMR (400 MHz, $CDCl_3$): δ = 7.68 (d, *J* = 8.4 Hz, 2H), 7.53 (d, *J* = 8.4 Hz, 2H), 6.75 (d, *J* = 4.2 Hz, 2H), 6.40 (d, *J* = 4.3 Hz, 2H), 3.11 (q, *J* = 7.6 Hz, 4H), 1.38 (t, *J* = 7.6 Hz, 6H); ^{13}C NMR (75 MHz, $CDCl_3$): δ = 164.3, 141.6, 135.4, 134.1, 132.5, 130.7, 130.4, 123.6, 117.8, 81.8, 75.9, 22.3, 13.0; ^{19}F NMR (376 MHz, $CDCl_3$): δ = 145.24 (q, *J* = 32.7 Hz); HRMS (ESI; positive): $[M]^+$ *m/z* calcd for $C_{42}H_{36}B_2F_4N_4Na$ 717.2962, found 717.2954.

Synthesis and characterization of ionic liquids

All ionic liquids were prepared according to literature procedures or modified literature procedures[2] following the general sequences shown below. All ionic liquids were purified as follows: ionic liquids were dissolved in CH_2Cl_2, followed by filtration to get rid of inorganic impurities. Next, ionic liquids were repeatedly treated with charcoal in EtOH at elevated temperatures followed by filtration and removal of EtOH in *vacuo* (for an azeotropic removal of residual water). Finally, the ionic liquids were dried under vacuum for 8-12 hours. All sample preparations and spectroscopic measurements were conducted immediately after removing the ionic liquids from the vacuum with care to minimize the exposure to moisture.

[C_4-mim]PF_6:[3]

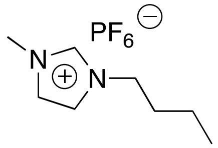

^{1}H NMR (300 MHz, DMSO-d_6): δ = 9.08 (s, 1H), 7.74 (s, 1H), 7.67 (s, 1H), 4.14 (t, *J* = 6.9 Hz, 2H), 3.83 (s, 3H), 1.75 (pent, *J* = 7.2 Hz, 2H), 1.24 (sext, *J* = 7.2 Hz, 2H), 0.89 (t, *J* = 7.2 Hz, 3H);

^{19}F NMR (282 MHz, DMSO-d_6): δ = –70.2 (d, *J* = 710.4 Hz)

[C_4-mim]NTf_2:[3]

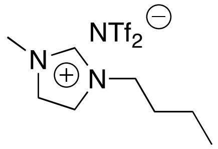

^{1}H NMR (300 MHz, DMSO-d_6): δ = 9.09 (s, 1H), 7.75 (t, *J* = 1.8 Hz, 1H), 7.68 (t, *J* = 1.8 Hz, 1H), 4.14 (t, *J* = 6.9 Hz, 2H), 3.83 (s, 3H), 1.75 (pent, *J* = 7.5 Hz, 2H), 1.24 (sext, *J* = 7.5 Hz, 2H), 0.89 (t, *J* = 7.2 Hz, 3H);

^{19}F NMR (282 MHz, DMSO-d_6): δ = –78.8 (s)

[C_{12}-mim]NTf_2:[4]

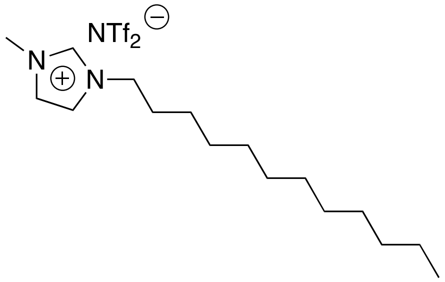

^{1}H NMR (300 MHz, DMSO-d_6): δ = 9.08 (s, 1H), 7.75 (s, 1H), 7.68 (s, 1H), 4.13 (t, *J* = 7.0 Hz, 2H), 3.83 (s, 3H), 1.75 (m, 2H), 1.22 (m, 18H), 0.84 (m, 3H);

^{19}F NMR (282 MHz, DMSO-d_6): δ = –78.8 (s)

Spectroscopic measurements

UV-Vis absorption and fluorescence spectra were obtained using a Cary 50 bio UV–visible Spectrophotometer (Varian Inc.) and Cary Eclipse Spectrofluorometer (Varian Inc.), respectively. All the measurements were done in 0.4mm x 1cm cuvettes with optical density below 0.05, unless mentioned otherwise. In order to measure the quantum yield, absorption spectra of **BD** was collected followed by measuring the integrated fluorescence intensity of the sample. A solution of fluorescein in 0.1M NaOH was used as a reference (quantum yield: 0.90). Fluorescence lifetime was measured on a FluoTime 300 fluorometer (PicoQuant, Inc.) using a 470 nm diode laser. The fluorometer was equipped with an ultrafast microchannel plate detector (MCP) from Hamamatsu, Inc. The fluorescence lifetimes were measured in the magic angle condition and data analyzed using FluoFit4 program from PicoQuant, Inc (Germany) using multi-exponential fitting model:

$$I(t) = \sum_i \alpha_i e^{-t/\tau_i} \qquad (1)$$

where, α_i is the fractional amplitude of the intensity decay of the ith component at time t and τ_i is the lifetime of the ith component. The amplitude and intensity weighted average lifetimes ($<\tau>_{amp}$, and $<\tau>_{int}$) were calculated using the following equations:

$$\langle\tau\rangle_{amp} = \frac{\sum_i \alpha_i \tau_i}{\sum_i \alpha_i} \qquad (2)$$

$$\langle\tau\rangle_{int} = \sum_i f_i \tau_i \quad where \quad f_i = \frac{\alpha_i \tau_i}{\sum_i \alpha_i \tau_i} \qquad (3)$$

And f_i represents fractional intensities for each fluorescence lifetime component.

Radiative and non-radiative rates were calculated using experimentally measured quantum yield and fluorescence lifetimes using the following equation:

$$\emptyset_f = \frac{k_r}{k_r + k_{nr}} \quad \text{where,} \quad \tau = \frac{1}{k_r + k_{nr}} \qquad (4)$$

The (normalized or reduced) χ^2 (in Tables S3 and S6) is the optimization parameter for least squares fitting analysis defined as:

$$\chi^2 = \frac{1}{n-p} \sum_{i=1}^{n} \left[\frac{I_i - I(t_i)}{\sigma_i} \right]^2$$

n is the number of data points, p is the number of freely varying parameters, σ_i is the standard deviation, I_i is the measured intensity at time t_i and $I(t_i)$ is the fitted theoretical value for the fitted intensity decay function at time t_i.[5]

Table S1. Photophysical properties (absorption maxima, emission maxima, fluorescence lifetime, and quantum yield) of dyes **1**, **2** and **BD** in molecular solvents at 20°C

DYE	SOLVENT	λ_{abs} / nm	λ_{ex} / nm	$\langle\tau\rangle_{int}$ / ns	$\langle\tau\rangle_{amp}$ / ns	Φ
**1**	ethanol	510	523	1.390	1.250	0.19
	propylene glycol	511	524	2.870	2.500	0.35
	glycerol	513	525	4.610	4.610	0.80
**2**	ethanol	512	529	0.550	0.290	0.04
	propylene glycol	515	530	0.870	0.810	0.13
	glycerol	515	525	3.060	2.820	0.48
**BD**	ethanol	512	532	0.326	0.223	0.04
	propylene glycol	513	532	1.463	1.344	0.19
	glycerol	516	533	4.500	4.155	0.48

Table S2. Effect of temperature on the viscosity of molecular solvents

SOLVENT	T / ^{o}C	Viscosity / mPa•s
ethanol (1985 ppm)[a]	5	1.8
	20	1.4
	35	1.0
	50	0.8
propylene glycol (1100 ppm)[a]	5	198
	20	70
	35	30
	50	15
glycerol (970 ppm)[a]	5	ND[b]
	20	1475
	35	506
	50	181

a – water content as determined by KF titration at room temperature;

b – not determined; m.p. of glycerol 17.8^{o}C.

Table S3. Effect of temperature on fluorescence lifetime of **BD** in molecular solvents

SOLVENT	T / °C	τ_1 / ns	τ_2 / ns	τ_3 / ns	α_1	α_2	α_3	$<\tau>_{int}$	$<\tau>_{amp}$	χ^2
EtOH	5	2.461	0.500	0.210	0.0022	0.1523	0.8455	0.343	0.262	0.95
	20	3.796	0.470	0.190	0.0012	0.1083	0.8905	0.326	0.223	0.97
	35	4.096	0.410	0.155	0.0012	0.0748	0.9240	0.303	0.178	0.95
	50	3.993	0.433	0.134	0.0012	0.0316	0.9672	0.287	0.148	0.99
Propylene glycol	5	3.514	2.087	-	0.1717	0.8283	-	2.456	2.332	0.95
	20	2.224	1.163	-	0.1714	0.8286	-	1.463	1.344	0.96
	35	1.759	0.719	-	0.0751	0.9249	-	0.891	0.797	1.03
	50	2.156	0.476	-	0.0160	0.9840	-	0.591	0.503	0.97
Glycerol	5	5.288	2.081	-	0.8366	0.1634	-	5.059	4.764	0.99
	20	4.701	1.573	-	0.8282	0.1718	-	4.500	4.155	0.99
	35	4.049	2.00	-	0.6439	0.3561	-	3.610	3.321	1.04
	50	4.045	1.848	-	0.2343	0.7657	-	2.729	2.363	1.02

Table S4. Effect of temperature on viscosity of ionic liquids

IONIC LIQUID	Temperature / °C	Viscosity / mPa•s
$[C_4\text{-mim}]PF_6$ (1720 ppm)[a]	5	1307
	10	880
	20	435
	30	234
	35	179
	40	139
	50	89
	60	61
$[C_4\text{-mim}]NTf_2$ (707 ppm)[a]	5	169
	10	129
	20	78
	30	51
	35	44
	40	36
	50	27
	60	21
$[C_{12}\text{-mim}]NTf_2$ (430 ppm)[a]	5	729
	10	516
	20	248
	30	144
	35	116
	40	97
	50	66
	60	47

a – water content as determined by KF titration at room temperature.

Table S5. Photophysical properties (absorption maxima, emission maxima, fluorescence lifetime, and quantum yield) of **BD** in ionic liquids at 20°C

DYE	SOLVENT	λ_{abs} / nm	λ_{ex} / nm	$<\tau>_{int}$ / ns	$<\tau>_{amp}$ / ns	Φ
**BD**	$[C_4\text{-mim}]PF_6$	514	533	2.244	2.101	0.29
	$[C_4\text{-mim}]NTf_2$	514	533	1.384	1.260	0.22
	$[C_{12}\text{-mim}]NTf_2$	516	536	2.276	1.906	0.48

Table S6. Effect of temperature on fluorescence lifetime of **BD** in ionic liquids

SOLVENT	T / °C	τ_1 / ns	τ_2 / ns	τ_3 / ns	α_1	α_2	α_3	$<\tau>_{int}$	$<\tau>_{amp}$	χ^2
[C_4-mim]PF_6	5	4.011	2.402	2.226	0.4758	0.4519	0.0723	3.366	3.154	1.03
	10	3.934	3.000	2.109	0.2218	0.2838	0.4944	2.951	2.764	0.99
	20	3.000	1.777	1.730	0.2731	0.5058	0.2210	2.244	2.101	0.99
	30	2.684	1.426	1.461	0.1234	0.7292	0.1474	1.684	1.583	0.95
	35	2.618	1.330	1.083	0.0646	0.7100	0.2255	1.442	1.344	1.02
	40	2.922	1.174	0.870	0.0023	0.7525	0.2257	1.224	1.146	0.99
	50	4.053	0.997	0.748	0.0054	0.3849	0.6097	0.933	0.862	1.03
	60	5.071	1.241	0.648	0.0029	0.0300	0.9670	0.764	0.679	0.98
[C_4-mim]NTf_2	5	4.148	2.213	1.548	0.0038	0.4622	0.5003	2.104	1.953	1.00
	10	4.934	1.991	1.334	0.0013	0.4546	0.5320	1.823	1.681	0.97
	20	5.812	1.819	1.120	0.0056	0.1617	0.8327	1.384	1.260	0.97
	30	6.750	1.814	0.873	0.0036	0.0496	0.9467	1.082	0.941	0.95
	35	6.219	1.850	0.756	0.0041	0.0248	0.9711	0.972	0.806	0.95
	40	5.981	1.681	0.660	0.0045	0.0183	0.9772	0.890	0.700	0.99
	50	5.765	1.402	0.522	0.0048	0.0144	0.9808	0.794	0.561	1.03
	60	5.486	0.974	0.424	0.0054	0.0199	0.9747	0.769	0.462	1.03

Table S6. Effect of temperature on fluorescence lifetime of **BD** in ionic liquids (cont'd)

SOLVENT	T / °C	τ_1 / ns	τ_2 / ns	τ_3 / ns	α_1	α_2	α_3	$<\tau>_{int}$	$<\tau>_{amp}$	χ^2
[C_{12}-mim]NTf_2	5	4.391	3.744	2.264	0.0950	0.1782	0.7268	2.951	2.730	1.02
	10	4.704	2.619	2.014	0.1136	0.2987	0.5877	2.778	2.500	1.02
	20	6.447	2.970	1.539	0.0206	0.1801	0.7992	2.276	1.906	0.99
	30	6.069	2.920	1.239	0.0243	0.0707	0.9050	1.944	1.486	0.98
	35	5.455	1.926	1.055	0.0344	0.0895	0.8761	1.815	1.285	0.97
	40	5.434	1.742	0.932	0.0343	0.0694	0.8963	1.752	1.153	0.99
	50	5.401	1.633	0.736	0.0312	0.0287	0.9401	1.627	0.907	1.02
	60	5.312	1.62	0.594	0.0295	0.0103	0.9602	1.610	0.744	0.98

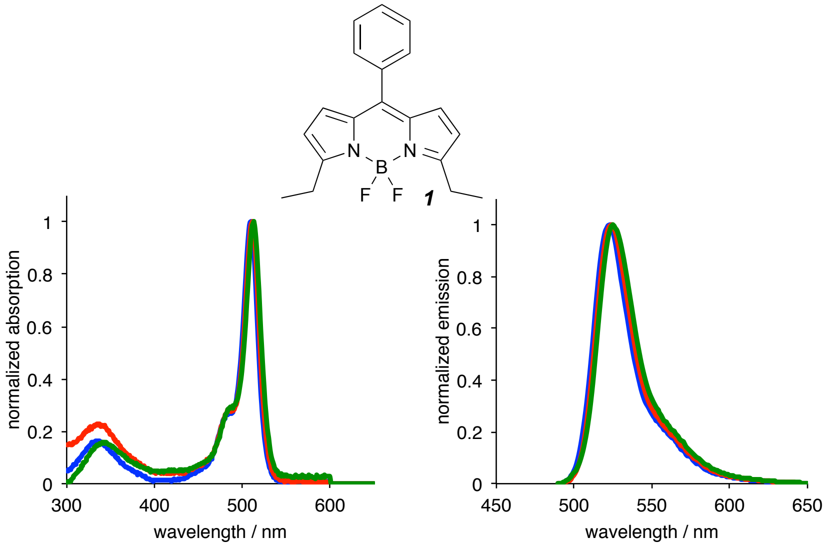

1 in ethanol
1 in propylene glycol
1 in glycerol

Figure S1. Absorption (left) and emission (right) spectra of dye **1** in molecular solvents at 20°C

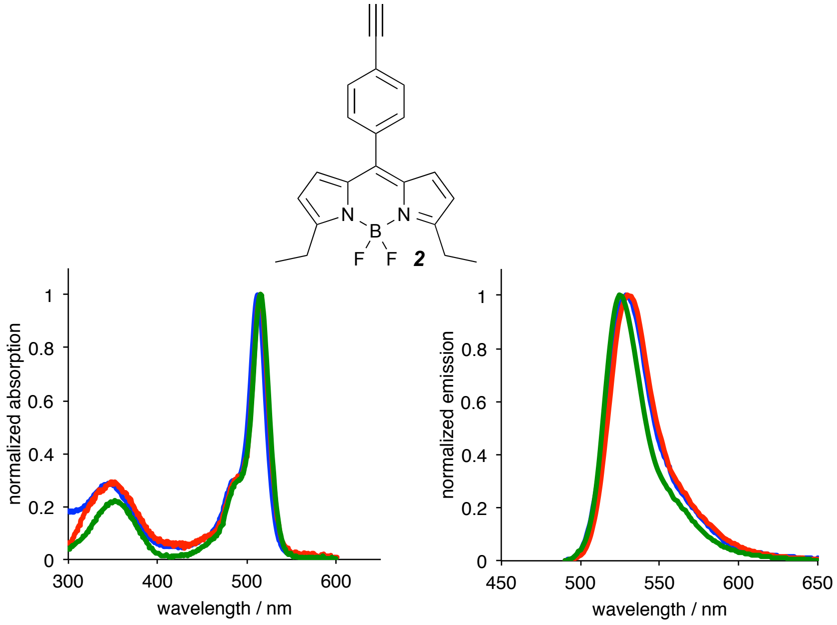

2 in ethanol
2 in propylene glycol
2 in glycerol

Figure S2. Absorption (left) and emission (right) spectra of dye **2** in molecular solvents at 20°C

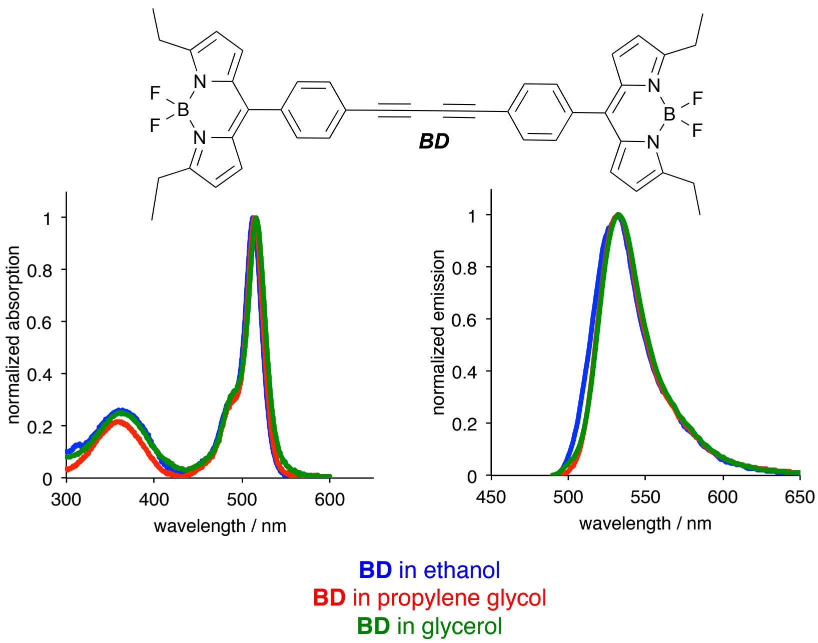

Figure S3. Absorption (left) and emission (right) spectra of dye **BD** in molecular solvents at 20°C

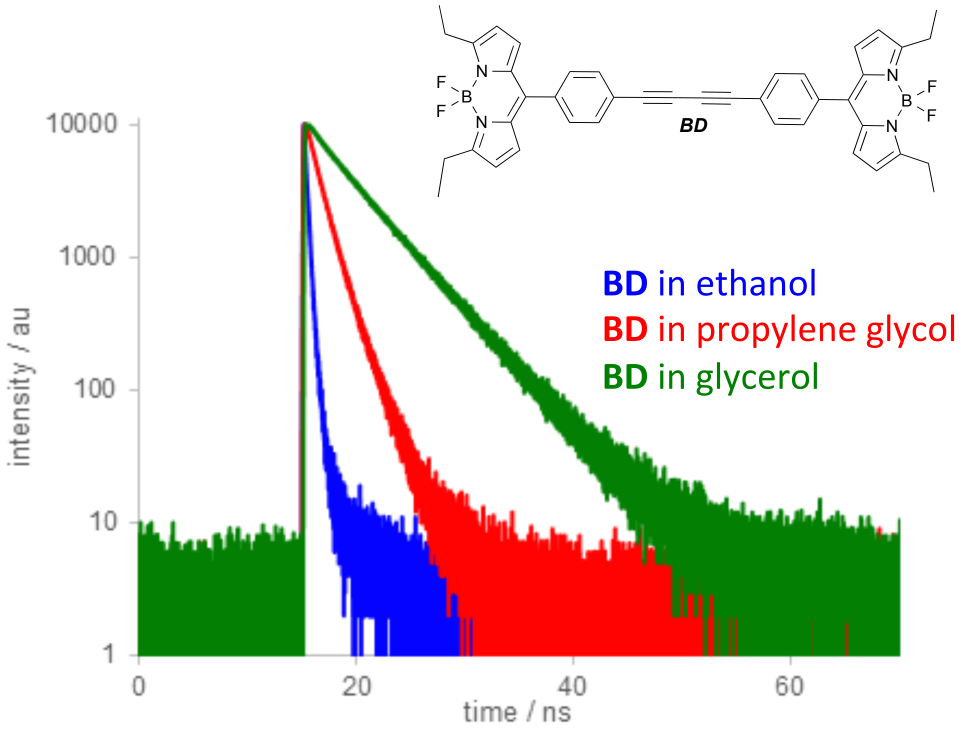

Figure S4. Fluorescence intensity decays for **BD** dye in molecular solvents at 20°C

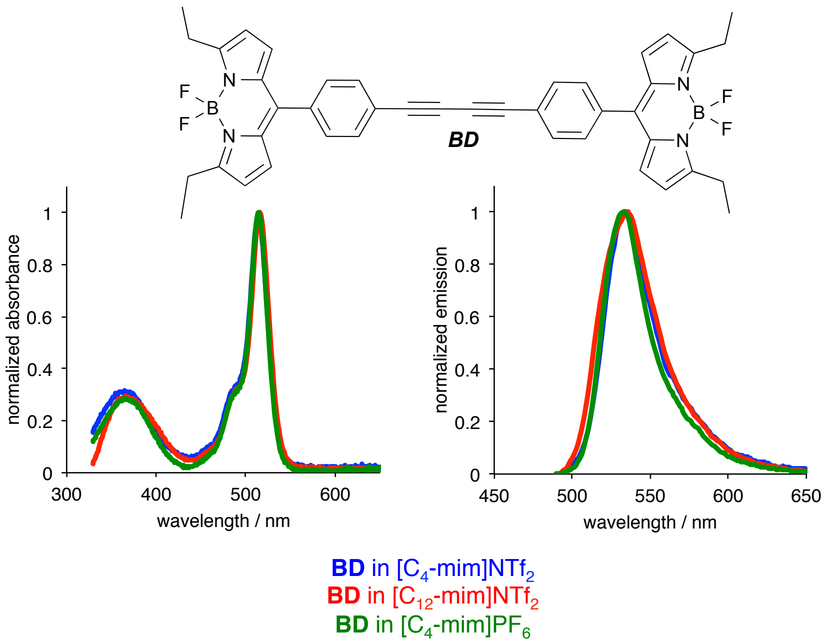

Figure S5. Absorption (left) and emission (right) spectra of dye **BD** in ionic liquids at 20°C

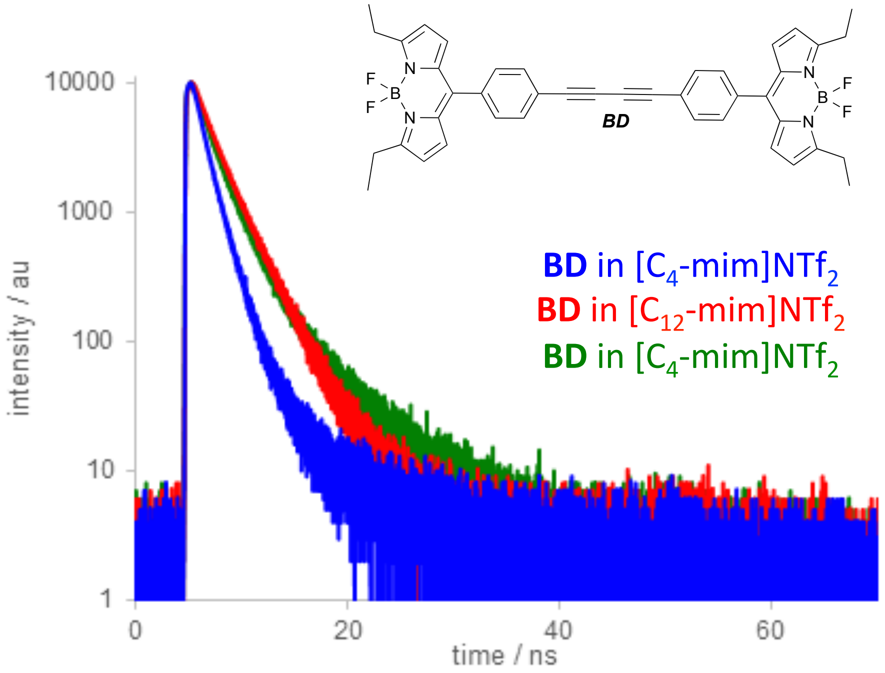

Figure S6. Fluorescent intensity decays for **BD** dye in ionic liquids at 20°C

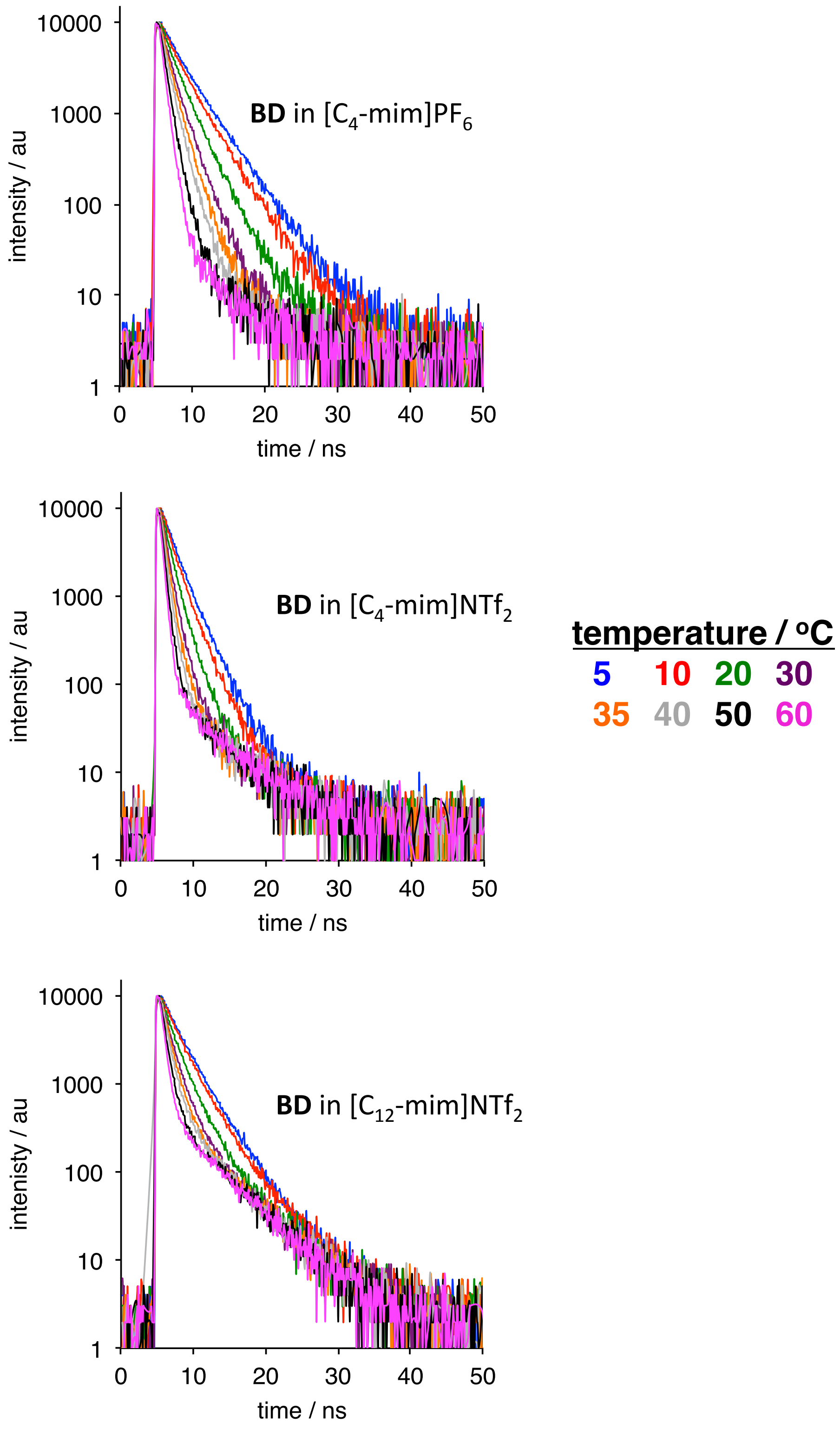

Figure S7. Temperature-dependent fluorescence intensity decays of **BD** in ionic liquids

References

1. (a) L. P. Jameson and S. V. Dzyuba, *Beilstein J. Org. Chem.,* 2013, **9**, 786; (b) N. W. Smith, A. Alonso, C. M. Brown and S. V. Dzyuba, *Biochem. Biophys. Res. Commun.,* 2010, **391**, 1455.

2. (a) J. G. Huddleston, H. D. Willauer, R. P. Swatloski, A. E. Visser and R. D. Rogers, *Chem. Commun*., 1998, 1765; (b) S. V. Dzyuba and R. A. Bartsch, *J. Heterocyclic Chem.,* 2001, **38**, 265; (c) S. V. Dzyuba, K. D. Kollar and S. S. Sabnis, *J. Chem. Ed.,* 2009, **86**, 856.

3. S. V. Dzyuba and R. A. Bartsch, *ChemPhysChem,* 2002, **3**, 161.

4. M. Blesic, M. H. Marques, N. V. Plechkova, K. R. Seddon, L. P. N. Rebelo and A. Lopes, *Green Chem.,* 2007, **9**, 481.

5. I. Gryczynski, R. Luchowski, S. Bharill, J. Borejdo, and Z. Gryczynski**.** “Nonlinear Curve-Fitting Methods for Time-Resolved Data Analysis.” Chapter 12. *FLIM Microscopy in Biology and Medicine*. A. Periasamy, R. M. Clegg (Eds), Chapman and Hall/CRC; **2009**, 472 p.
